# The LIM-Homeodomain transcription factor Islet-1 is required for the development of sympathetic neurons and adrenal chromaffin cells

**DOI:** 10.1016/j.ydbio.2013.04.027

**Published:** 2013-05-03

**Authors:** Katrin Huber, Priyanka Narasimhan, Stella Shtukmaster, Dietmar Pfeifer, Sylvia M. Evans, Yunfu Sun

**Affiliations:** a Institute for Anatomy and Cell Biology, Department of Molecular Embryology, Albert-Ludwigs-University, Freiburg, Germany; b Department of Hematology and Oncology, University Medical Center, Freiburg, Germany; c Department of Medicine, University of California San Diego, 9500 Gilman Drive, La Jolla, CA 92093, USA; d Shanghai East Hospital, Tongji University School of Medicine, 150 Jimo Road, Shanghai 200120, China

**Keywords:** Islet-1, Sympathoadrenal cell lineage, Sympathetic neuron, Chromaffin cell, Mouse

## Abstract

Islet-1 is a LIM-Homeodomain transcription factor with important functions for the development of distinct neuronal and non-neuronal cell populations. We show here that Islet-1 acts genetically downstream of Phox2B in cells of the sympathoadrenal cell lineage and that the development of sympathetic neurons and chromaffin cells is impaired in mouse embryos with a conditional deletion of Islet-1 controlled by the wnt1 promotor. Islet-1 is not essential for the initial differentiation of sympathoadrenal cells, as indicated by the correct expression of pan-neuronal and catecholaminergic subtype specific genes in primary sympathetic ganglia of Islet-1 deficient mouse embryos. However, our data indicate that the subsequent survival of sympathetic neuron precursors and their differentiation towards TrkA expressing neurons depends on Islet-1 function. In contrast to spinal sensory neurons, sympathetic neurons of Islet-1 deficient mice did not display ectopic expression of genes normally present in the CNS. In Islet-1 deficient mouse embryos the numbers of chromaffin cells were only mildly reduced, in contrast to that of sympathetic neurons, but the initiation of the adrenaline synthesizing enzyme PNMT was abrogated and the expression level of chromogranin A was diminished. Microarray analysis revealed that developing chromaffin cells of Islet-1 deficient mice displayed normal expression levels of TH, DBH and the transcription factors Phox2B, Mash-1, Hand2, Gata3 and Insm1, but the expression levels of the transcription factors Gata2 and Hand1, and AP-2β were significantly reduced. Together our data indicate that Islet-1 is not essentially required for the initial differentiation of sympathoadrenal cells, but has an important function for the correct subsequent development of sympathetic neurons and chromaffin cells.

## Introduction

The neural crest is a transient embryonic structure that gives rise to many different cells types, including neuronal and glial cells of the peripheral nervous system, endocrine cells of the adrenal medulla, thyroid C cells, and melanocytes (for review see Le Douarin and Kalcheim (1999)). During the past decade considerable progress has been made in deciphering the molecular networks underlying the segregation, differentiation and maturation of neural crest derived cells.

Sympathetic neurons of the para- and pre-vertebral ganglia and the endocrine adrenal chromaffin cells originate from the trunk neural crest. Though distinct in function, they share many characteristics as they both possess the machinery to synthesize, store and release the catecholamines noradrenaline (and adrenaline in a sub-population of chromaffin cells). Originally both cell types were thought to be derived from a common bi-potential catecholaminergic sympathoadrenal (SA) precursor, that develops from neural crest cells in the primary sympathetic ganglia close to the dorsal aorta (Anderson et al., 1991; Anderson and Axel, 1986). Though recent studies have suggested that the neuronal and endocrine SA subline-age may segregate before the onset of catecholaminergic differentiation, both nevertheless employ similar early developmental programs to establish the traits of catecholamine releasing cells (for review see Huber (2006) and Huber et al. (2009)).

This is reflected by their expression of a common set of transcription factors, including the homeodomain transcription factors Phox2A and B, the basic helix-loop-helix transcription factors Mash-1 and Hand2 and the zinc-finger transcription factor Gata3 and Insm1. These transcription factors are induced by BMP −2/4/7 and can be first detected shortly after the primary sympathetic ganglia have formed from a subset of neural crest cells that have migrated to the vicinity of the dorsal aorta (for a recent review see Rohrer (2011)). Phox2B has been identified as master regulator of SA cell differentiation (Pattyn et al., 1999). Its loss leads to the lack of expression of any genes characteristic for neuronal and catecholaminergic function and it abrogates the expression of the above mentioned transcription factors apart from Mash-1, which is prematurely downregulated (Hendershot et al., 2008; Huber et al., 2005; Pattyn et al., 1999; Tsarovina et al., 2008; Wildner et al., 2008). Mash-1 (Guillemot et al., 1993; Hirsch et al., 1998; Pattyn et al., 2006), Hand2 (Morikawa et al., 2007; Hendershot et al., 2008; Schmidt et al., 2009), Gata3 (Moriguchi et al., 2006; Tsarovina et al., 2008) and Insm1 (Wildner et al., 2008) have more discrete functions and together with Phox2B they act as a network rather than in linear cascade to promote the differentiation of SA cells. Loss of function studies have revealed that during sympathetic neuron development they control catecholaminergic and generic neuronal differentiation, proliferation of progenitors and immature neurons, survival of postmitotic neurons (Gata3) and maintenance of differentiated sympathetic neuron properties (Hand2, Phox2B, for review see Rohrer (2011)). Similar, but not identical functions of this gene regulatory network have been proposed for the development of adrenal chromaffin cells (for review see Huber et al. (2009)).

The LIM-Homeodomain transcription factor Islet-1 is expressed in distinct neuronal and non-neuronal populations and it is known as a key regulator of the development of spinal motoneurons (Pfaff et al., 1996), the pancreas (Ahlgren etal., 1997)and theheart(Cai etal., 2003). Multiple aspects of motoneuron development depend on Islet-1, including specification (Pfaff et al., 1996; Song et al., 2009), axonal growth (Liang et al., 2011) and patterning (Kania and Jessell, 2003). In the peripheral nervous system Islet-1 is widely expressed, including sensory neurons of the dorsal root ganglia (DRG) and sympathetic ganglia (Avivi and Goldstein, 1999; Sun et al., 2008). Loss of function studies have revealed an essential role of Islet-1 for the developmentof the sensory neurons of the dorsal root ganglia, with the TrkA-positive nociceptive neurons being most prominently affected by Islet-1 deficiency. While the early development of dorsal root ganglia appeared grossly normal following ablation of Islet-1 in the neural crest, later in development a selective loss of nociceptive markers and enhanced apoptosis was observed in dorsal root ganglia. Gene expression analysis further revealed a prolonged expression of transcription factors that are normally restricted to early sensory neurogenesis and ectopic expression of genes that are normally present in the CNS, but not in sensory ganglia (Sun et al., 2008). Islet-1 has been suggested to regulate sensory neuron gene expression epistatically with Brn3A through binding to an enhancer in the neurod4 gene (Dykes et al., 2011). Direct transcriptional downstream targets of Islet-1 also include Mef2C (Dodou et al., 2004) and Hb9 (Lee and Pfaff, 2003), which are important for heart and motoneuron differentiation, respectively.

Though the expression of Islet-1 in developing sympathetic neurons is well documented, its function has remained unclear up to date. In this study we have investigated the role of Islet-1 for the development of sympathetic neurons and chromaffin cells. Our data indicate that Islet-1 acts genetically downstream of Phox2B and does not essentially affect the initial differentiation of SA cells, but is required for subsequent survival and correct maturation of sympathetic neurons and chromaffin cells.

## Materials and methods

### Experimental animals

The generation and genotyping of wnt1cre conditional Islet-1 deficient mice containing a Rosa26 LacZ reporter gene (Sun et al., 2008) and Phox2B^Lacz^ mice (Pattyn et al., 1999) were described previously. Embryos were staged considering midday of the day of the vaginal plug as embryonic day 0.5. Embryos with the genotype Islet-1^flox/flox^/wnt1cre are referred to as Islet-1^wnt1cre^ mouse embryos and embryos with the genotype Islet-1^flox/+^/wnt1cre are referred to as control mouse embryos.

### Histology

Pregnant mice were killed by cervical dislocation. Embryos were removed from the uterus, briefly rinsed with phosphate-buffer (PB, pH 7.4) and fixed in PB containing 4% paraformaldehyde (PFA) overnight. Tissues were then rinsed 3 times with PB and transferred into 30% sucrose in PB for cryoprotection. After immersion in sucrose overnight the tissue was coated with OCT^™^ compound (Tissue Tek), frozen on a metal bridge that was placed in liquid nitrogen, and stored at −70 °C until further processing. Tissues were then cut into 10 μm serial sections, mounted on Superfrost^™^ slides, and air dried for 30 min, before performing in situ hybridization, immunfluorescence staining or TdT dUTP nick end labeling analysis, respectively.

### In situ hybridization

In situ hybridization on cryosections and preparation of digoxigenin-labeled probes for mouse Mash-1 (Guillemot and Joyner, 1993), mouse Phox2B (Pattyn et al., 1997), mouse Phox2A (Valarché et al., 1993), mouse Hand2 (Srivastava et al., 1997), mouse Gata3 (Ko et al., 1991), mouse Gata2 (Kala et al., 2009), mouse Insm1 (Wildner et al., 2008), mouse chromogranin A (Wildner et al., 2008), mouse neurofilament-68 (NF; Huber et al., 2002), mouse SCG10 (Gut et al., 2005), mouse tyrosine hydroxylase (TH; Zhou et al., 1995), mouse DBH (Tiveron et al., 1996), mouse c-Ret (Pachnis et al., 1993), mouse PNMT (Cole et al., 1995), LacZ (Huber et al., 2005), mouse LHX1 and LHX2 (Abellan et al., 2010) and mouse LBXCOR 1 (Glassmann et al., 2009) were performed as described previously (Ernsberger et al., 1997). Mouse Islet-1 (bp: 351–1053 of NM_021459.4), mouse TrkA (bp 1443–2163 of NM_001033124.1) and mouse TrkC (bp 466–1161 of NM_182809.2) were cloned by RT-PCR using a pGEM-T vector system (Promega) following the manufacturer’s instruction.

### Immunofiuorescence staining

Antibodies and immunoreagents were obtained from the following sources and diluted as indicated in brackets: polyclonal sheep anti-tyrosine hydroxylase (TH, 1:500; Millipore GMBH, Schwalbach Germany), rabbit anti-phospho-histone H3 (pH3, 1:1000, Millipore GMBH, Schwalbach Germany), rabbit anti-Phox2B (1:200, kindly provided by Drs. Jean Francois Brunet and Christo Goridis, IBDM, Marseille, France), biotinylated donkey-anti-sheep antibody (1:200), Cy2^™^-conjugated donkey-anti-rabbit antibody (1:200) and Cy3^™^-conjugated streptavidin (1:500) were obtained from Dianova, Hamburg, Germany.

For immunfluorescence-staining sections were pretreated with 10% serum corresponding to the secondary antibody in PBS containing 0.1% Triton X-100. Primary antibodies were incubated overnight at 4 °C. After rinsing in PBS sections were incubated with Cy2^™^-conjugated or biotinylated secondary antibody for 2 h at room temperature. Sections were then rinsed in PBS, incubated with Cy3^™^-conjugated streptavidin (in case a biotinylated secondary antibody was used) and after final rinsing in PBS mounted with Fluoro-Mount containing DAPI (Dianova, Hamburg, Germany).

### TdT dUTP nick end labeling (TUNEL) analysis

For detection of apoptotic cells we performed TUNEL on 10 μm cryosections using an ApoTag^™^ In Situ APOPTOSIS Detection Kit (Millipore) following the manufacturer’s instructions. Subsequently, TH-immunofluorescence staining was carried out as described above. TUNEL positive cells were counted in every 10th section through the thoracic sympathetic chain within the TH-immunoreactive area. The size of the TH immunoreactive area was subsequently measured by using ImageJ and the number of TUNEL positive cells per TH immunoreactive area was determined.

### Electronmicroscopy

For electronmicroscopy, adrenals from E16.5 embryos were fixed by immersion in a mixture of glutaraldehyde (1.5%) and paraformaldehyde (1.5%) in 0.1 M phosphate buffer (PB) at pH 7.4 for 48 h and rinsed several times with PB. Organs were then postfixed in 1% OsO_4_ in PB, rinsed in PB and block-stained with 1% uranyl acetate. After dehydration through increasing concentrations of ethanol, the tissue was embedded in Durcupan (Fluka). Ultrathin sections (50 nm) were examined with a Zeiss EM10.

### Microarray analysis

Whole adrenal glands of E14.5 control and Islet-1^wnt1cre^ mouse embryos were dissected and stored in RNAlater® (Qiagen, Düsseldorf, Germany) at −80 °C until further processing. After genotyping adrenal glands were pooled (four adrenal glands per sample, four samples per group) and homogenized in Trizol. Total RNA was extracted from the homogenized tissue according to manufacturer’s protocol and eluted in RNAse free water. Microrarray analysis was performed using mouse Gene ST 1.0 Arrays (Affymetrix UK, High Wycombe, UK) as previously described (Spittau et al., 2013).

### Statistical analysis

To assess the statistical significance a Student’s *t*-test for a two-tailed distribution and a two-sample unequal variance was performed.

## Results

### Expression of Islet-1 in SA cells

**Fig. 1:**
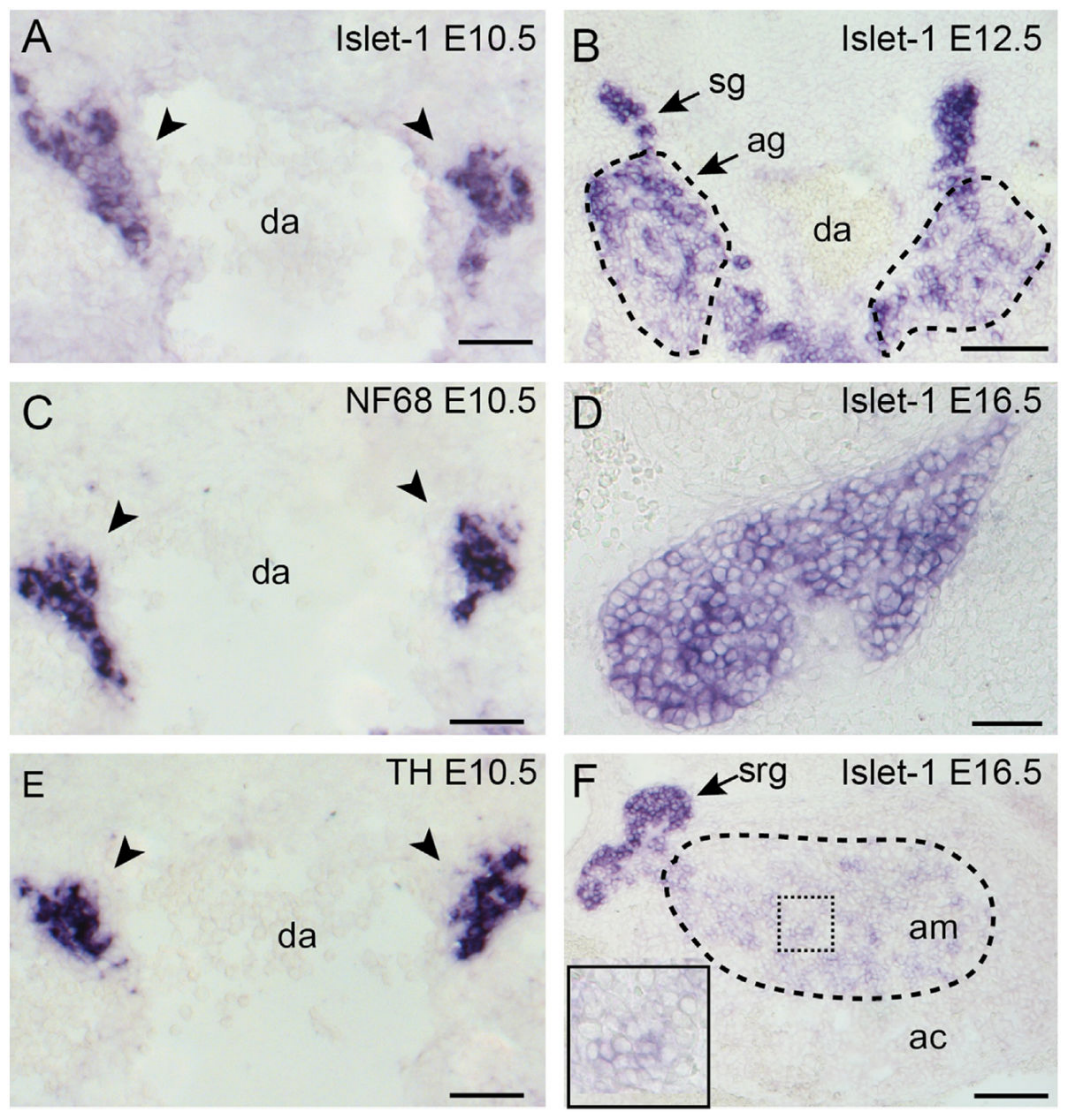
Islet-1 is expressed in primary sympathetic ganglia (arrowheads) of E10.5 mouse embryos (A). In situ hybridizations for NF68 (C) and TH (E) were performed on near adjacent sections. In the developing adrenal gland Islet-1 can be detected at E12.5 (B). To identify the area of the developing adrenal gland the adreno-cortical marker Sf1 was used as a marker on an adjacent section (not shown). Islet-1 expression can be detected at least until E16.5 in sympathetic ganglia (D, thoracic sympathetic ganglion) and in adrenal chromaffin cells (F) albeit at lower levels. Insert: higher magnification. The dashed line indicates the border of the adrenal gland anlage in (B) and the adrenal medulla in (F). (ac) Adrenal cortex, (ag) adrenal gland anlage, (am) adrenal medulla (da), dorsal aorta, (sg) sympathetic ganglion, (srg) suprarenal ganglion; bars: (A, B, C, E, and F) 100 μm, (D) 50 μm.

We first investigated the expression of Islet-1 in developing mouse sympathetic ganglia and adrenal glands. Islet-1 expression is initiated in the primary sympathetic ganglia around E9.5–E10 (not shown) and can be detected throughout all axial levels by E10.5 (Fig. 1A). The expression of Islet-1 is maintained in sympathetic ganglia at least until E16.5 (Fig. 1D).

Adrenal Chromaffin cells share a common origin from the neural crest with sympathetic neurons and express a similar set of genes during their early development. We could detect Islet-1 expression in the developing adrenal gland as soon as it is colonized by chromaffin progenitors at E12.5 (Fig. 1B). Islet-1 expression is maintained in the adrenal medulla at least until E16.5, albeit at lower levels than in sympathetic ganglia (Fig. 1F).

### Islet-1 expression in SA cells depends on Phox2B

**Fig. 2:**
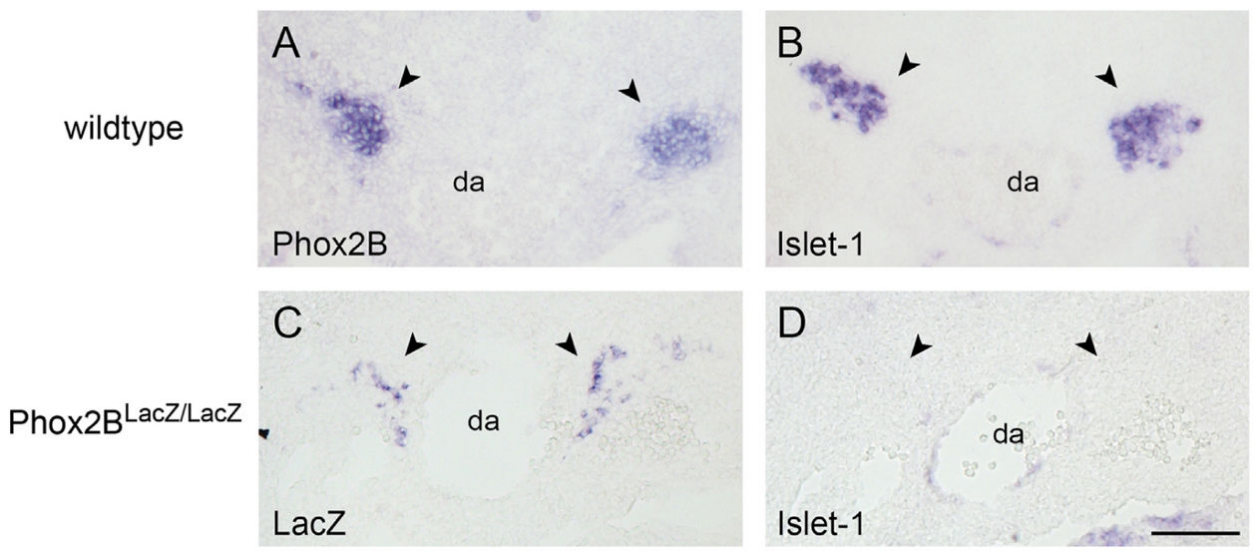
Islet-1 expression is lacking in the area of sympathetic ganglia of Phox2B^LacZ/LacZ^ mice at E11.5. Photomicrographs show cross sections through thoracic sympathetic ganglia (arrowheads) of E11.5 wildtype (A and B) and Phox2B^LacZ/LacZ^ mouse embryos (C and D). In-situ-hybridizations for Phox2B, LacZ and Islet-1 were performed in near adjacent sections. (da) Dorsal aorta. Bar: 100 μm.

The homeodomain transcription factor Phox2B controls the development of distinct neuronal populations and it is associated with catecholaminergic differentiation as well as the establishment of autonomic reflex pathways (Dauger et al., 2003; Pattyn et al., 1999, 2000). The differentiation of SA cells crucially depends on Phox2B (Huber et al., 2005; Pattyn et al., 1999) and most other components of the transcription factor network that governs the development of SA cells, including Hand2, Phox2A and Insm1, act downstream of Phox2B (for review see Huber (2006) and Rohrer (2011)). To explore, whether Phox2B is acting upstream of Islet-1 in the SA lineage we analyzed Islet-1 expression in Phox2B^Lacz/Lacz^ mouse embryos. As anticipated, Islet-1 expression was not detectable in LacZ positive cells accumulating at the dorsal aorta of E11.5 Phox2B^LacZ/LacZ^ embryos (Fig. 2), indicating that Islet-1 acts genetically downstream of Phox2B in the SA lineage.

### The initial differentiation of SA cells does not essentially depend on Islet-1

In order to define a putative role of Islet-1 for the development of SA cells we employed Islet-1^wnt1cre^ mice. This mouse line has been shown to efficiently eliminate the expression of exon 3/4 of the Islet-1 transcript in neural crest derivatives, as previously described (Sun et al., 2008). Due to the early onset of wnt1 expression in the neural plate before the delamination of neural crest cells (Echelard et al., 1994) wnt1cre mediated excision of Islet-1 should occur before the expression of Islet-1 is initiated in cells of the SA lineage.

**Fig. 3:**
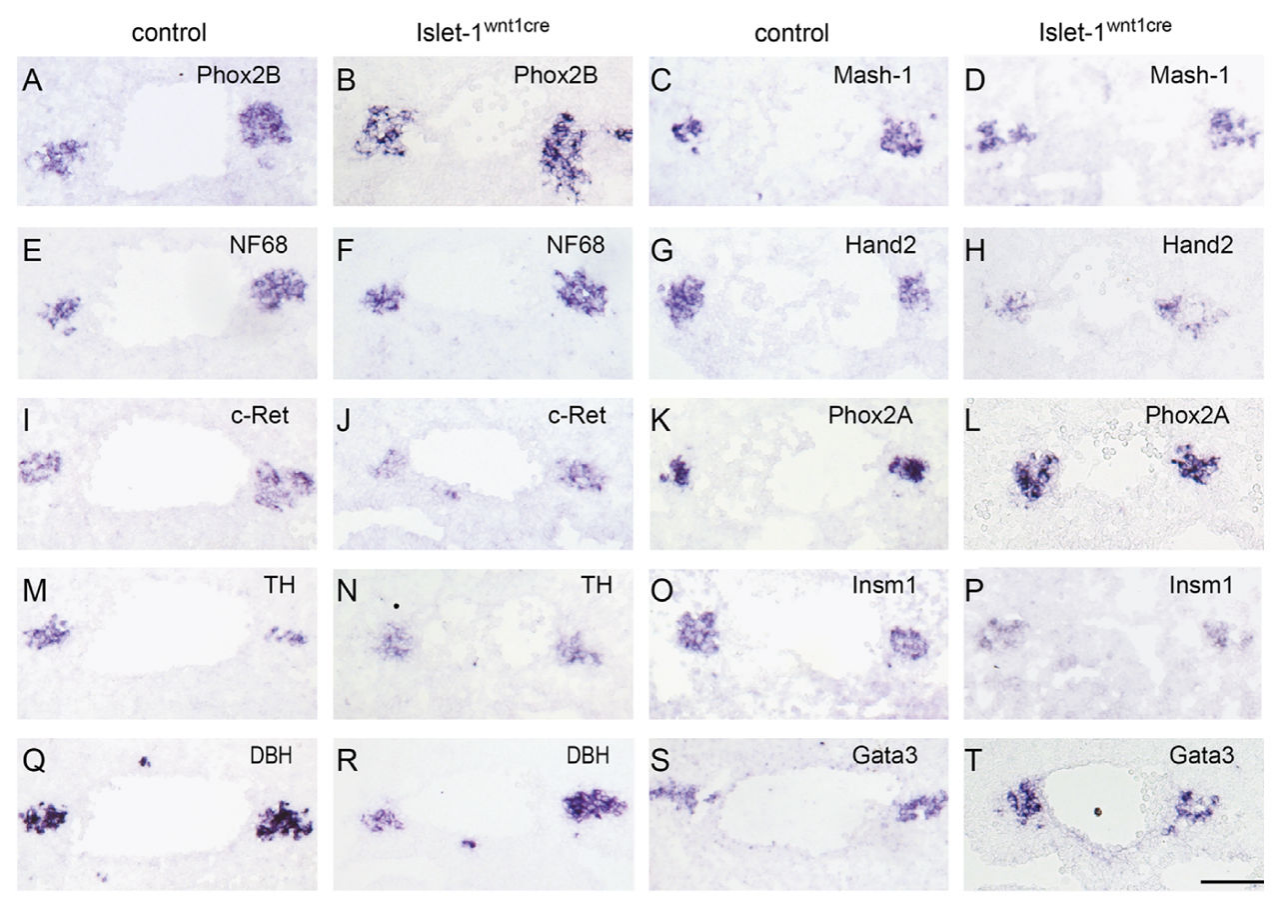
The initial differentiation of SA cells is grossly normal in Islet-1^wnt1cre^ mouse embryos. Photomicrographs show sections through thoracic primary sympathetic ganglia of control (A, C, E, G, I, K, M, O, Q, and S) and Islet-1^wnt1cre^ (B, D, F, H, J, L, N, P, R, and T) mouse embryos at E10.5. In situ hybridizations were performed for Phox2B (A and B), neurofilament 68 (E and F), c-Ret (I and J), TH (M and N), DBH (Q and R), Mash1 (C and D), Hand2 (G and H), Phox2A (K and L), Insm1 (O and P) and Gata3 (S and T). Bar: 100 μm.

The size of primary sympathetic ganglia of Islet-1^wnt1cre^ mouse embryos appeared grossly normal at E10.5, using Phox2B (Fig. 3A and B) or LacZ (not shown) as a marker. In order to identify a putative impact of Islet-1 on the initial differentiation of SA cells we analyzed the expression of the transcription factors Mash-1, Hand2, Phox2A, Gata3, and Insm1 as well as genes indicative of neuronal and catecholaminergic differentiation, i.e. neurofilament (NF) 68, SCG10 (not shown), tyrosine-hydroxylase (TH, the rate limiting enzyme for catecholamine synthesis), dopamine-β-hydroxylase (DBH, the enzyme that converts dopamine into noradrenaline) and the proto-oncogene c-ret in primary sympathetic ganglia of E10.5 Islet-1^wnt1cre^ mouse embryos and control littermates (Fig. 3A–T). This analysis did not reveal any striking differences between control and Islet-1^wnt1cre^ mouse, suggesting that Islet-1 is not essentially required for the initial differentiation of SA cells. Furthermore, in contrast to the sensory neurons of the dorsal root ganglia (Sun et al., 2008), sympathetic neurons of Islet-1 deficient mice did not display ectopic expression of genes that are normally present in the CNS like LHX1, LHX2 and LBXCOR1 at E10.5, E11.5 (not shown) or E12.5 (see Supplementary Fig. S1).

### Sympathetic ganglia of Islet-1^wnt1cre^ mice become progressively atrophic

**Fig. 4:**
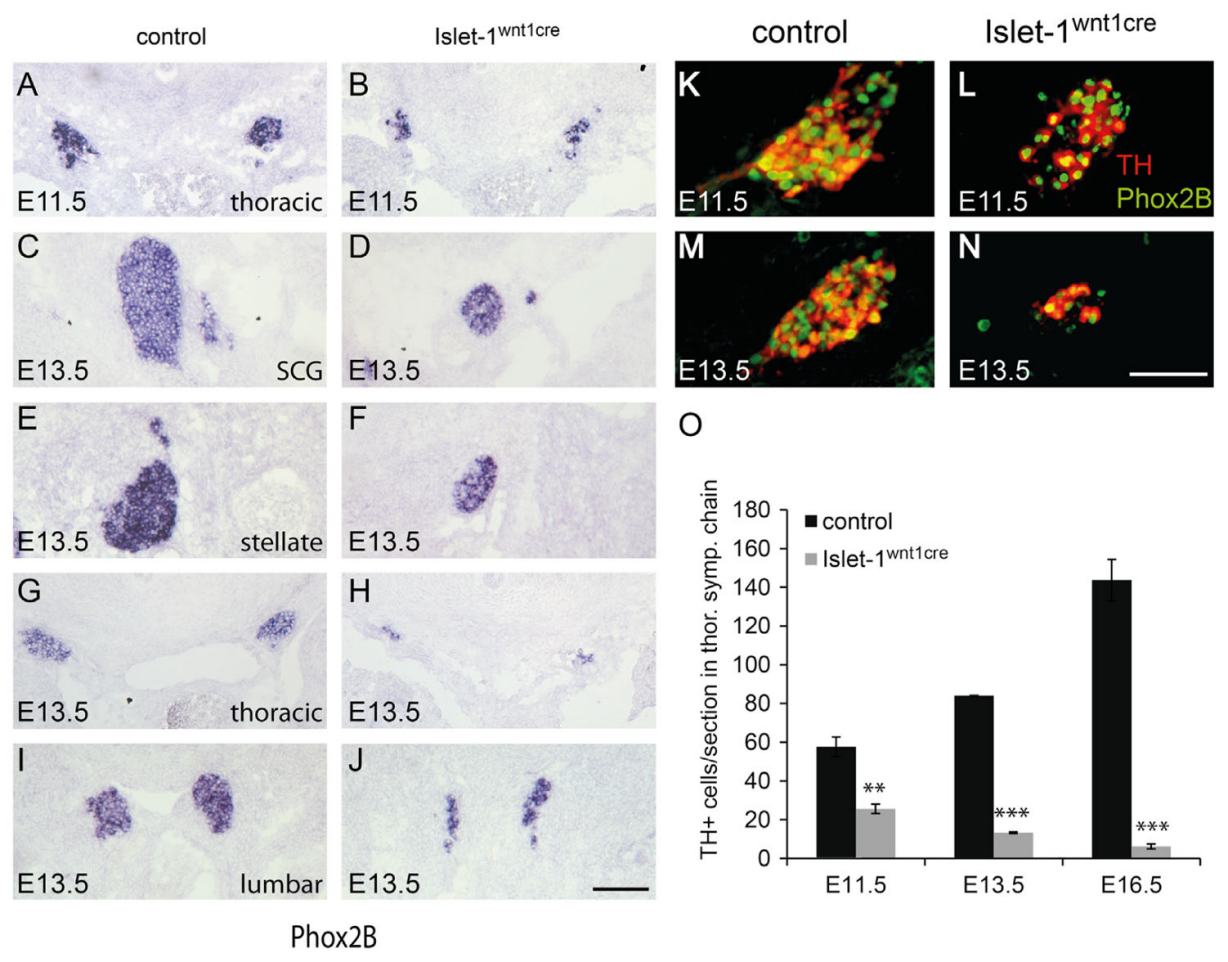
Sympathetic ganglia of Islet-1^wnt1cre^ mouse embryos become progressively atrophic. The size of sympathetic ganglia, as visualized by Phox2B ISH, is reduced in Islet-1^wnt1cre^ mouse embryos (B, D, F, H, and J) as compared to control embryos (A, C, E, G, and I) at E11.5 (A and B) and E13.5 (C–J). (K–N) Double immunofluorescence-staining using antibodies against TH (red cytoplasmic stain) and Phox2B (green nuclear stain) of sympathetic ganglia of control and Islet-1^wnt1cre^ mouse embryos at E11.5 (K and L) and E13.5 (M and N). Note that in both control and Islet-1^wnt1cre^ mouse embryos virtually all Phox2B-immunoreactive cells are also positive for TH. (O) Counts of TH-immunoreactive cells in the thoracic sympathetic chain of control and Islet-1^wnt1cre^ mouse embryos. Data represent the number of TH positive cells per section and are presented as means±s.e.m. Every 10th section throughout the thoracic sympathetic chain of at least 3 embryos per group was analyzed. Bars: (A–J) 100 μm, (K–N) 50 μm. ^**^p<0.01; ^***^p<0.01.

At E11.5 and later stages the size of sympathetic ganglia of Islet-1^wnt1cre^ mouse embryos appeared considerably smaller as compared to control littermates. This could be observed throughout all axial levels and regardless whether Phox2B (Fig. 4A–J), LacZ, NF68 or TH (Fig. 4K–N) was used as a marker. Like in control mouse embryos, the vast majority of Phox2B positive cells in sympathetic ganglia of Islet-1^wnt1cre^ mouse embryos were also TH-immunoreactive at E11.5 and E13.5 (Fig. 4K–N), indicating that the transition of Phox2B+/TH− progenitors to Phox2B+/TH+ SA cells was not significantly impaired in Islet-1 deficient mice. In order to quantify the effect of Islet-1 deficiency on sympathetic neuron numbers, we determined the number of TH-positive cells throughout the thoracic sympathetic chain in every 10th section (Fig. 4O). This analysis revealed a progressive reduction of the relative number of TH positive cells in sympathetic ganglia of Islet-1^wnt1cre^ mouse embryos as compared to control mouse embryos from E11.5 (amounting to 44% of control values) to E16.5 (amounting to 4% of control values).

**Fig. 5:**
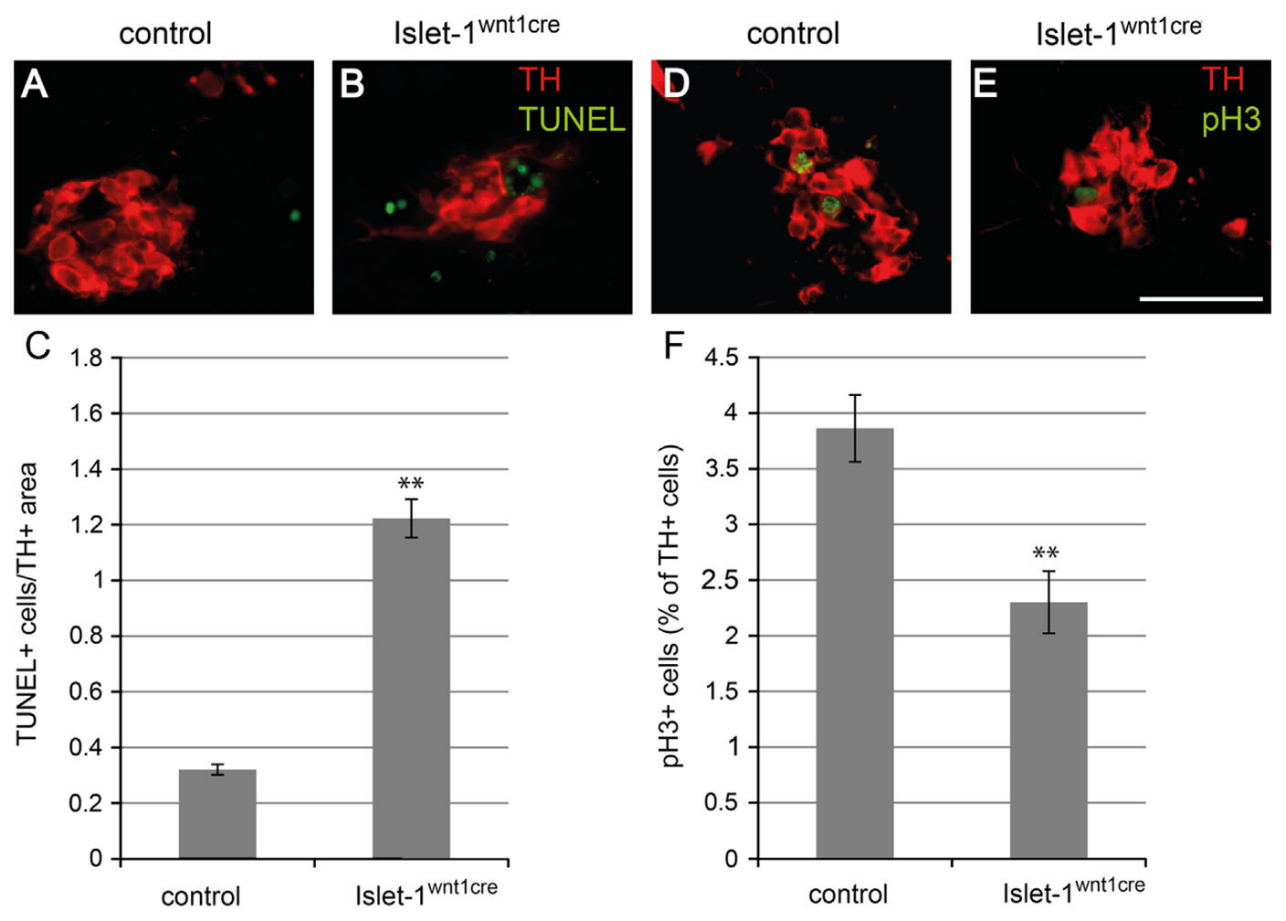
Apoptosis is enhanced and proliferation is reduced in sympathetic ganglia of Islet-1^wnt1cre^ mouse embryos at E11.5. (A and B) Photomicrographs showing TUNEL (green) and TH-immunofluorescence-staining (red) of sympathetic ganglia of a control (A) and an Islet-1^wnt1cre^ mouse embryo (B) at E11.5. (C) Relative number of TUNEL-positive cells per TH-positive area in thoracic sympathetic ganglia of control and Islet-1^wnt1cre^ mouse embryos. (D and E) Double immunofluorescence-staining using antibodies against TH (red) and phosphohistone 3 (pH3, green) of sympathetic ganglia of control (D) and Islet-1^wnt1cre^ mouse embryos (E). (F) Number of pH3- positive cells (% of TH positive cells) in thoracic sympathetic ganglia of control and Islet-1^wnt1cre^ mouse embryos. Data are presented as means±s.e.m. Every 10th section throughout the thoracic sympathetic chain of at least 3 embryos per group was analyzed. Bar: 50 μm. ^**^p<0.01.

To determine the cause of the cell number reduction in sympathetic ganglia of Islet-1^wnt1cre^ mouse embryos we performed TUNEL analysis and assessed the proliferative activity in sympathetic ganglia by using the M-phase mitotic marker pH3. As demonstrated in (Fig. 5A–C we observed a significant increase of the number of TUNEL positive cells in the area of developing sympathetic ganglia of E11.5 Islet-1^wnt1cre^ mouse embryos as compared to control mouse embryos. In addition the percentage of TH positive cells that were also positive for pH3 was diminished in Islet-1^wnt1cre^ mouse embryos (Fig. 5D–F). Thus enhanced cell death and reduced proliferation account for the reduced size of sympathetic ganglia.

### The expression of TrkA and c-Ret is altered in sympathetic ganglia of Islet-1^wnt1cre^ mice

**Fig. 6:**
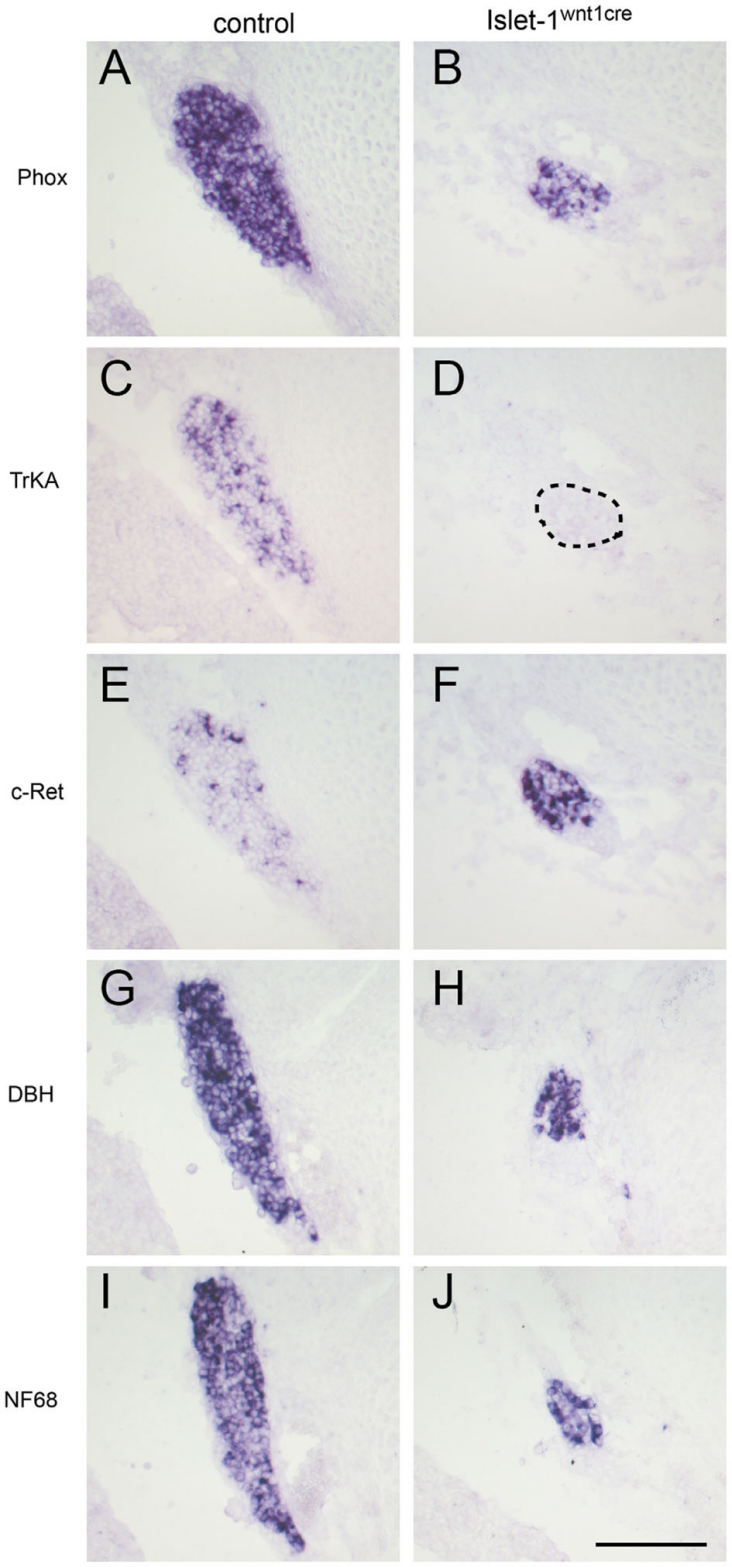
In situ hybridization for Phox2B (A and B) and TrkA (C and D), c-Ret (E and F), DBH (G and H) and neurofilament 68 (I and J) on sections through the thoracic sympathetic chain of E16.5 control and Islet-1^wnt1cre^ mouse embryos. Note that the majority of cells in control sympathetic ganglia express TrkA and a small subpopulation of cells expresses high levels of c-Ret. In contrast the few remaining sympathetic neurons in Islet-1^wnt1cre^ mouse embryos lack TrkA and uniformly express high levels of c-Ret, while the expression levels of DBH and neurofilament 68 appear unaltered. The area of the sympathetic ganglion is demarcated by a dashed line in (D). Bar: 100 μm.

At E16.5 we could still detect small clusters of sympathetic neurons in a few sections throughout the whole sympathetic chain of Islet-1^wnt1cre^ mouse embryos. These cells were LacZ positive (not shown) and still displayed apparently normal expression of Phox2B (Fig. 6A and B), DBH (Fig. 6G and H), NF 68 (Fig. 6I and J), TH, SCG10, Phox2A, Hand2, and Gata3 (not shown).

Accordingly the expression of Mash-1, which is transiently expressed in early developing sympathetic ganglia, had virtually disappeared in both control and Islet-1^wnt1cre^ mouse embryos by E16.5 (Supplementary Fig. S2) and it was unaltered at other developmental stages investigated (E10.5–E16.5). Furthermore, in contrast to spinal sensory neurons, sympathetic neurons of Islet-1^wnt1cre^ mouse embryos did not show an apparent upregulation of Insm1 expression at any age investigated (E10.5–E16.5, not shown).

Around E13.5 sympathetic neurons initiate the expression of the high-affinity NGF-receptor TrkA, which has an important function for their subsequent survival (Fagan et al., 1996). As shown in Fig. 6C, TrkA was expressed in the majority of sympathetic neurons of E16.5 control mouse embryos, while sympathetic neurons of Islet-1^wnt1cre^ mouse embryos lacked the expression TrkA (Fig. 6D). In contrast, the expression of TrkC, which precedes that of TrkA in sympathetic ganglia (Birren et al., 1993; Fagan et al., 1996) was not apparently affected in Islet-1^wnt1cre^ mouse embryos at the ages investigated (E11.5, E12.5, E13.5 and E16.5, see Supplementary Fig. S3 for E12.5).

The proto-oncogene c-Ret is a receptor typosine-kinase, which mediates signal transduction of members of the glial-cell line derived neurotrophic factor (GDNF) superfamily (for review see Airaksinen and Saarma (2002)). It is expressed homogenously in SA cells that aggregate at the dorsal aorta, but later in development its expression becomes restricted to the cholinergic sub-population of sympathetic neurons (Burau et al., 2004; Pachnis et al., 1993). As shown in Fig. 6E in E16.5 control mouse embryos high expression of c-Ret is limited to a subpopulation of sympathetic neurons, while the residual sympathetic neurons in Islet-1^wnt1cre^ mouse embryos display homogenously high expression of c-Ret (Fig. 6F). These results suggest that the few remaining cells in sympathetic ganglia of Islet-1^wnt1cre^ mice might not have matured correctly or that a TrkA negative, c-Ret positive subpopulation of sympathetic neurons is selectively spared in Islet-1^wnt1cre^ mouse embryos. Since the cholinergic subpopulation of sympathetic neurons expresses c-Ret, we performed in situ hybridization using probes for the cholinergic markers vesicular acetylcholine transporter (VAChT) and choline acetyltransferase (ChAT) (not shown). Our findings did not support the notion that the cholinergic subpopulation of sympathetic neurons specifically survives in Islet-1^wnt1cre^ mouse embryos.

### The correct maturation of chromaffin cells is impaired in Islet-1^wnt1cre^ mouse embryos

**Fig. 7:**
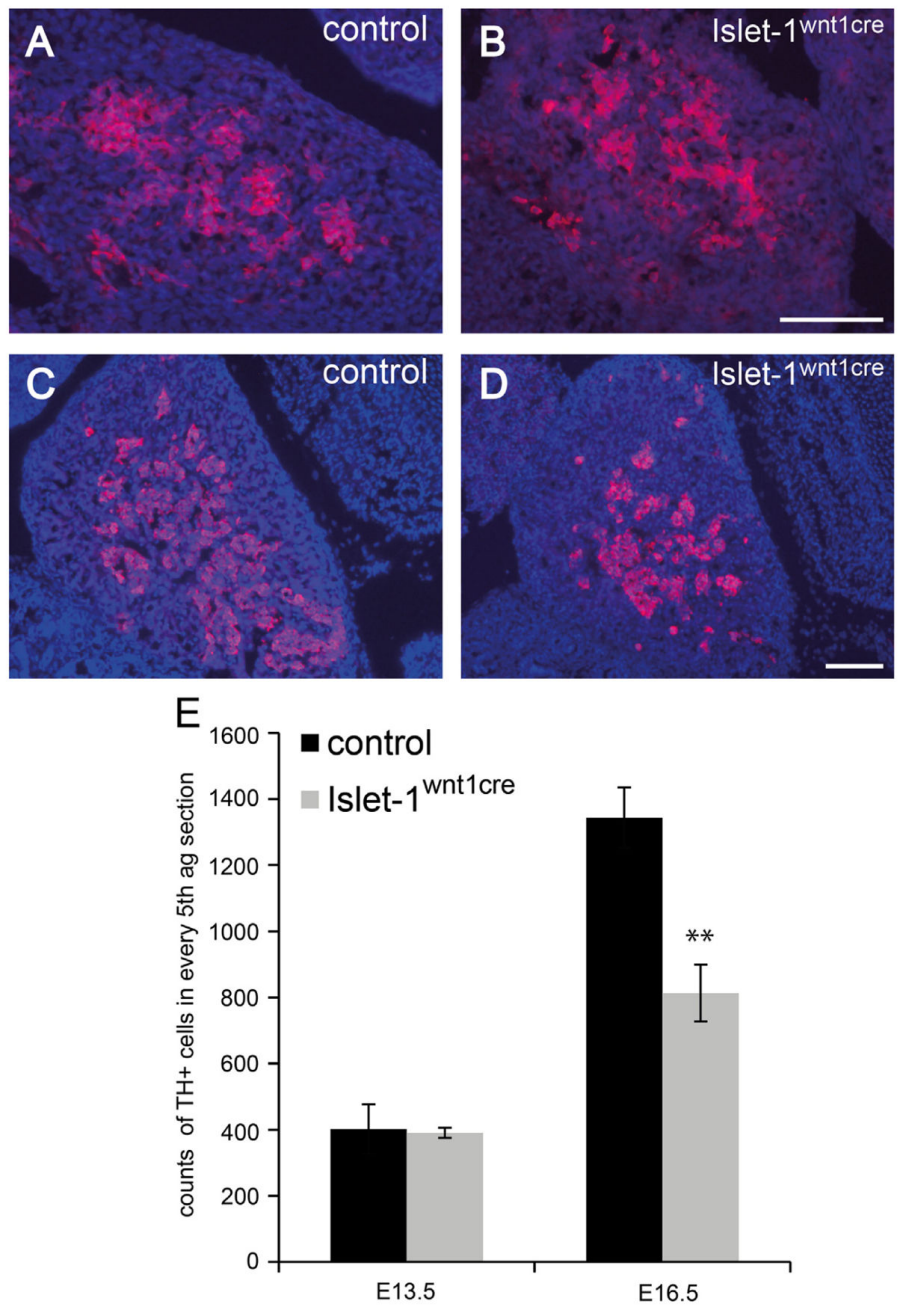
Photomicrographs showing cross sections through the adrenal glands of control (A and C) and Islet-1^wnt1cre^ mouse embryos (B and D) at E13.5 (A and B), and E16.5 (C and D) stained with an antibody against TH (red cytoplasmatic stain) and DAPI (blue nuclear stain). (E) Counts of TH-immunoreactive cells in sections of the adrenal glands of Islet-1^wnt1cre^ mouse embryos and control littermates. Data are presented as means±s.e.m. Every 5th section of at least 6 adrenal glands of 3 different animals for each group has been analyzed. Bars: 100 μm. ^**^p<0.01.

**Fig. 8:**
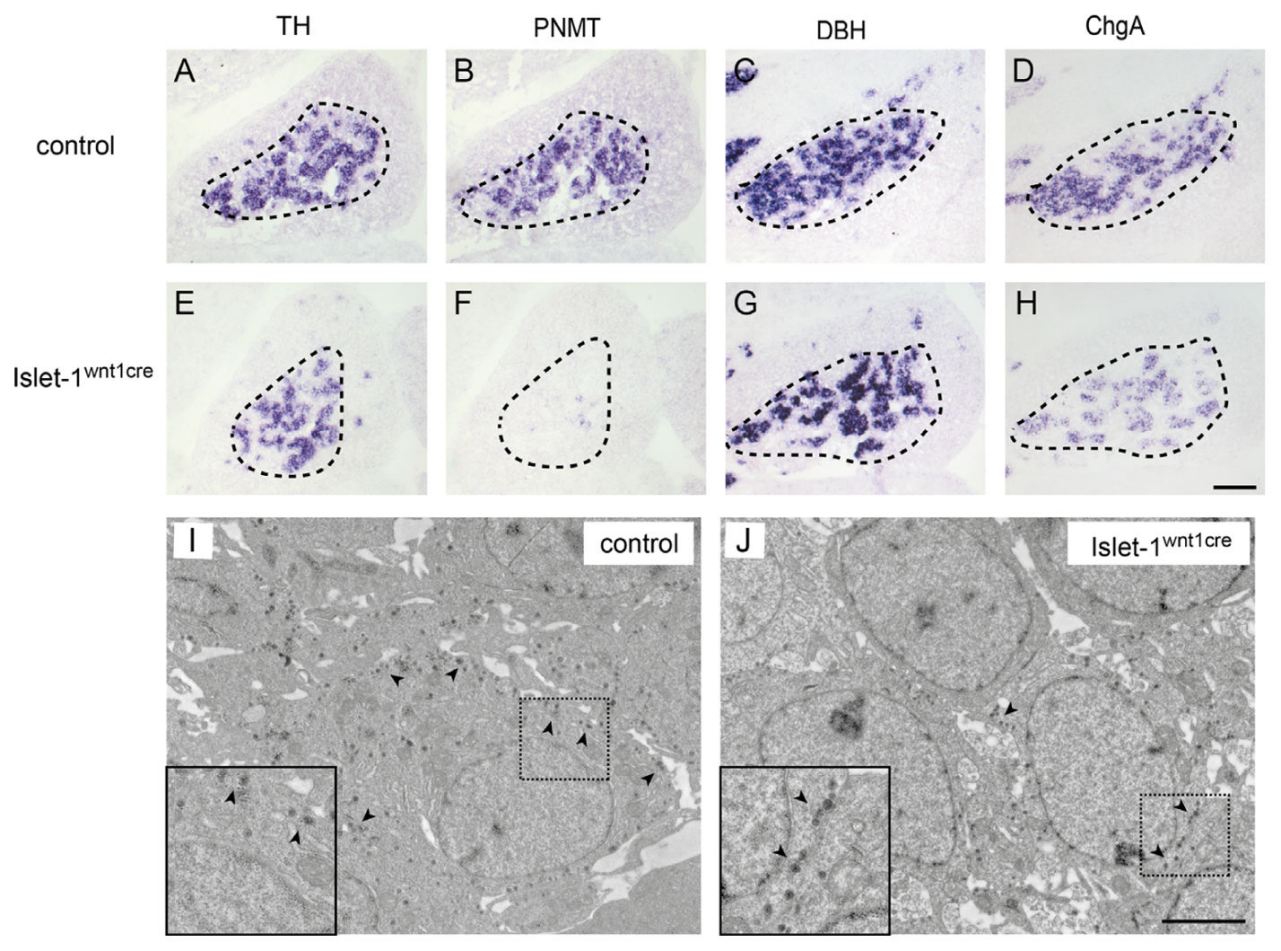
In situ hybridization for TH (A and E), PNMT (B and F), DBH (C and G) and chromogranin A (D and H) on sections of the adrenal glands of E16.5 control (A–D) and Islet-1^wnt1cre^ mouse embryos (E–H). Note that in the adrenal gland of Islet-1^wnt1cre^ mouse embryos the expression of PNMT is virtually lacking and expression levels of chromogranin A appear reduced. The approximate border of the adrenal medulla is demarcated by a dashed line. (I and J) Electronmicrographs of adrenal medullae of E16.5 control (I) and Islet-1^wnt1cre^ mouse embryos (J). SA cells in the adrenal gland of Islet-1^wnt1cre^ mouse embryos have undergone grossly normal chromaffin cell differentiation as indicated by the presence of chromaffin granules (arrowheads); insert: higher magnification. Bars: (A–H) 100 μm and (I and J) 2 μm.

In contrast to the massive atrophy of sympathetic ganglia, the numbers of adrenal chromaffin cells in Islet-1^wnt1cre^ mouse embryos as identified by TH-immunoreactivity were unaltered at E13.5 and relatively mildly affected at E16.5, amounting to 60% of control values (Fig. 7E). In situ hybridizations for TH (Fig. 8A and E) and DBH (C and G) did not reveal an apparent alteration of the expression of these enzymes in the adrenal medullae of Islet-1 deficient embryos at E16.5.

As chromaffin cell precursors mature, the adrenergic subpopulation initiates the expression of the enzyme phenyl-N-methyl-transferase (PNMT) that converts noradrenaline into adrenaline. As demonstrated in Fig. 8B, substantial numbers of chromaffin cells expressed PNMT in control embryos at E16.5. Strikingly, PNMT expression was virtually absent in the adrenal medullae of Islet-1^wnt1cre^ mouse embryos (Fig. 8F). In addition the expression of chromogranin (Chg) A, a secretory protein located in chromaffin vesicles, appeared diminished (Fig. 8D and H). These data suggest that some aspects of chromaffin cell maturation were impaired in Islet-1^wnt1cre^ mouse embryos. To assess the overall differentiation of chromaffin cells in Islet-1^wnt1cre^ mouse embryos we analyzed their ultrastructure. As shown in Fig. 8J adrenal medullary cells of Islet-1 deficient mice like control littermates displayed the typical secretory chromaffin granules in their cytoplasm (Coupland, 1972; Coupland and Tomlinson, 1989) indicating that the acquisition of an endocrine cell phenotype was not substantially impaired.

### Microarray analysis

To better understand the genetic program that is controlled by Islet-1 in cells of the SA lineage we performed gene expression profiling by microarray analysis. As stated above, sympathetic ganglia were substantially reduced in size from E11.5 onwards. Since dissection of sympathetic ganglia prior to E11.5 was not feasible, we focused on the adrenal gland. Microarray analysis was performed on RNA extracted from whole adrenal glands of Islet-1^wnt1cre^ and control mouse embryos obtained at E14.5, the age when PNMT expression is initiated in mouse chromaffin cells (Lohr et al., 2006). As summarized in Table 1 the overall alterations of gene expression levels were relatively mild and the only mRNA transcript showing a more than 3 fold change was that of the PNMT gene (13 fold down regulation). In contrast the expression levels of TH, DBH, Gata3, Mash-1, Phox2A, Phox2B and Insm1 were not significantly altered, consistent with the observation that chromaffin cell numbers were normal at early developmental ages and corroborating the finding that Islet-1 does not significantly influence the initial differentiation of SA cells.

However, the expression levels of some other transcription factors that are associated with SA cell development were reduced in the adrenal glands of Islet-1^wnt1cre^ mouse embryos, i.e. Gata2 (2.9 fold), Hand1 (2 fold) and AP-2β (2.5 fold). In cells of the SA lineage Gata2 and Hand1 act downstream of Gata3 and Hand2, respectively (Tsarovina et al., 2004; Vincentz et al., 2012). AP-2β, in contrast, is already expressed in migrating neural crest cells and it is required for sympathetic neuron survival and the maturation of chromaffin cells.

**Fig. 9:**
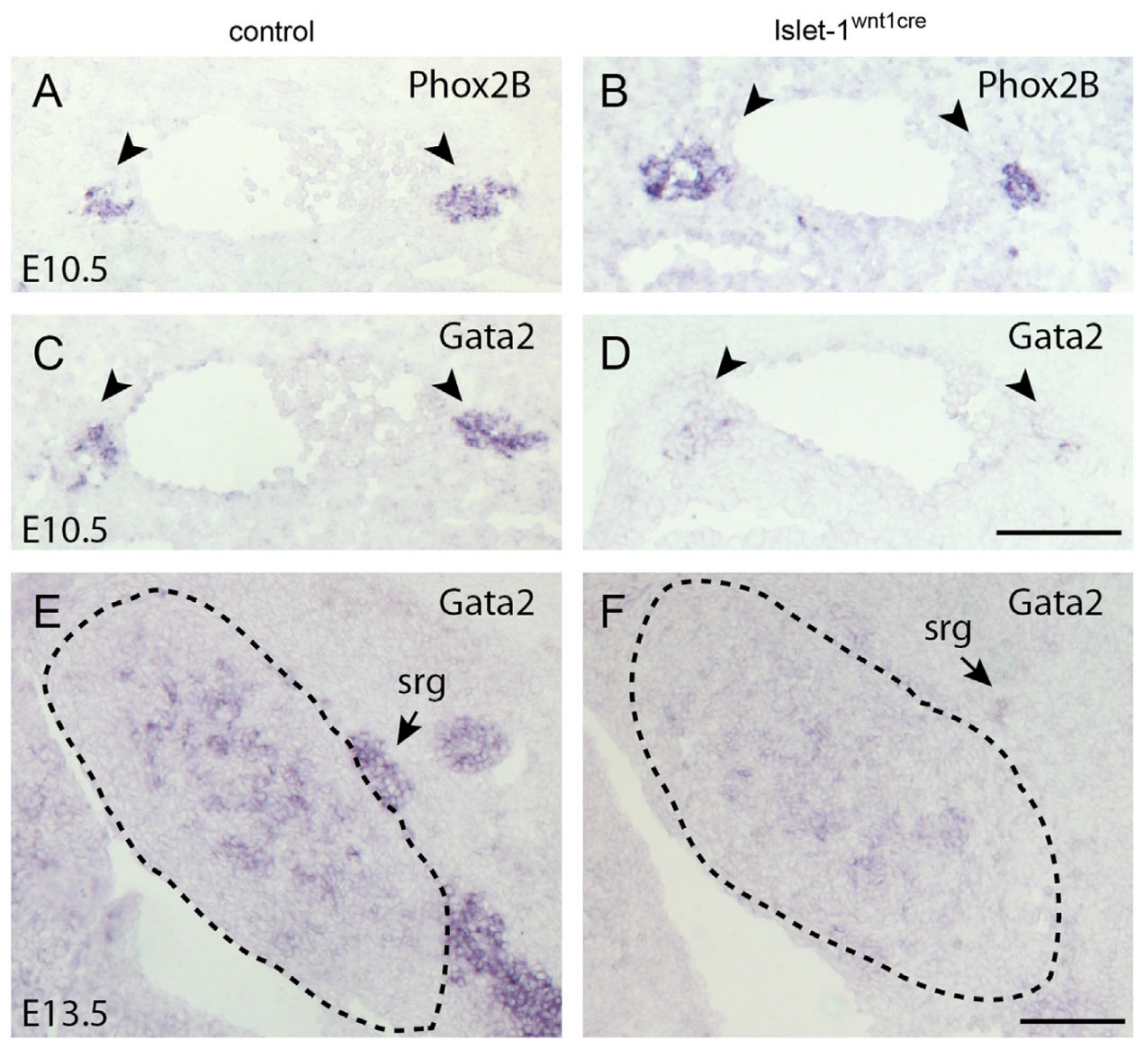
(A–D) In situ hybridization for Gata2 on sections through thoracic primary sympathetic ganglia (arrowheads) of E10.5 control (C) and Islet-1^wnt1cre^ mouse embryos (D). Phox2B in situ hybridizations (A and B) have been carried out on adjacent sections. (E and F) In situ hybridization for Gata2 on sections of the adrenal gland of E13.5 control (E) and Islet-1^wnt1cre^ embryos (F). The border of the adrenal gland is demarcated by a dashed line. Bars: 100 μm.

We assessed the expression of these transcription factors in primary sympathetic ganglia and the adrenal glands of Islet-1^wnt1cre^ mouse embryos and control littermates by in situ hybridization. While we did not obtain unequivocal results regarding the expression of Hand1 and AP-2β, we could show that Gata2 expression is markedly diminished in sympathetic ganglia of E10.5 Islet-1^wnt1cre^ mouse embryos (Fig. 9C and D). Likewise at E13.5 Gata2 expression was apparently reduced in the adrenal gland and the remaining sympathetic neurons of Islet-1^wnt1cre^ mouse embryos as compared to control littermates (Fig. 9E and F). These findings show that Islet-1 deficiency affects the expression levels of distinct molecular markers and transcription factors in chromaffin cells, including PNMT and Gata2.

## Discussion

### Islet-1 is required for the development of sympathetic neurons and chromaffin cells

The development of the nervous system is controlled by a complex network of intrinsic and extrinsic signals that govern cell fate specification, choice of neurotransmitter identity and axonal patterning. Islet-1 is a LIM-Homeodomain (LIM-HD) transcription factor, initially identified as a protein binding to the insulin gene enhancer (Karlsson et al., 1990). It regulates gene expression by binding via its homeodomain to the consensus sequence YTAATGR, while it can recruit co-factors mainly via the Limdomain (for review see Hobert and Westphal (2000)).

Islet-1 is required for the development of distinct neuronal populations in the peripheral and central nervous system, but it has also important functions outside the nervous system, e.g. during the development of the heart and the pancreas (Ahlgren et al., 1997; Cai et al., 2003). Neuronal populations that require Islet-1 for their development include spinal motoneurons (Pfaff et al., 1996), sensory neurons of the DRG (Sun et al., 2008), cholinergic forebrain neurons (Elshatory and Gan, 2008), and retinal ganglion cells (Elshatory et al., 2007). In the spinal cord specific combinations of LIM-HD proteins are considered to constitute a so called “Lim-code” that determines cell fate and axonal patterning (for review see Lumsden, 1995; Sockanathan, 2003). Gene ablation studies have revealed that Islet-1 is required for the initial generation of motoneurons as well as for multiple aspects of their subsequent development, including axonal pathfinding of specific subsets of motoneurons (Kania and Jessell, 2003; Liang et al., 2011; Pfaff et al., 1996).

In this study we show that Islet-1 also has important functions for the correct development of the derivatives of the SA cell lineage, i.e. sympathetic neurons and the endocrine adrenal chromaffin cells. Our data indicate that the initial differentiation of SA cells and the onset of catecholaminergic and neuronal differentiation does not essentially depend on Islet-1. Yet, in Islet-1^wnt1cre^ mouse embryos the survival and proliferation of sympathetic neuroblasts were impaired, resulting in massive atrophy of the sympathetic ganglia from E11.5 onwards. The few sympathetic neurons surviving up to E16.5 had apparently not matured correctly, as they lacked the high affinity NGF-receptor TrkA. In contrast to the severe loss of sympathetic neurons, chromaffin cell numbers in the adrenal gland of Islet-1^wnt1cre^ mouse embryos were relatively mildly reduced and the expression levels of the noradrenaline synthesizing enzymes TH and DBH were normal. However, the adrenaline synthesizing enzyme PNMT was virtually absent and chromogranin A expression levels were reduced, indicating a role of Islet-1 in establishing in more mature chromaffin cell traits. Thus, this study establishes Islet-1 as a transcription factor that acts relatively late during the differentiation of cells derived from the SA cell lineage.

### The role of Islet-1 for the development of sympathetic neurons versus DRG neurons

Like sympathetic neurons the sensory neurons of the dorsal root ganglia are neuronal derivatives of the neural crest. Loss of Islet-1 has been shown to predominantly affect the nociceptive TrkA-positive subpopulation of sensory neurons and the dorsal root ganglia of Islet-1 deficient mice exhibited enhanced apoptosis, similar to our observation in sympathetic ganglia (Sun et al., 2008). Apart from this, our study revealed profound differences between the developmental programs regulated by Islet-1 in sympathetic and sensory neurons. While sensory neurons of Islet-1 deficient mouse embryos displayed ectopic expression of genes specific for subpopulations of CNS neurons, this could not be observed in sympathetic ganglia. Furthermore, in DRG neurons the lack of Islet-1 leads to a prolonged expression of genes normally restricted to early sensory neurogenesis, as e.g. the basic helix-loop-helix transcription factors NeuroD4 and neurogenin1 (Sun et al., 2008). In contrast, as shown in this study, sympathetic neurons of Islet-1^wnt1cre^ mouse embryos normally downregulated Mash-1, a proneural transcription factor transiently expressed during their early development. The zinc-finger transcription factor Insm1 is expressed in both sensory and sympathetic neurons as well as in the endocrine pancreatic Islet cells, which also require Islet-1 for their development (Ahlgren et al., 1997). Interestingly, Insm1 showed enhanced expression in sensory but not in sympathetic neurons. Together these data imply that distinct gene regulatory programs are addressed by Islet-1 in sensory neuronal and sympathoadrenal neural crest derivatives and they underscore the context specificity of Islet-1 action as well as intrinsic differences between these neural crest derivatives.

### Islet-1 and the sympathoadrenal transcriptional network

The differentiation of SA cells is controlled by a cross-regulatory network of transcription factors including Phox2A/B, Mash1, Gata3, Hand2 and Insm1. The components of this network jointly control the expression of generic and subtype-specific neuronal genes, but they also reciprocally regulate their own expression. Phox2B is a master regulator of the development of SA cells and it is essentially required for the expression of all members of this network, apart from MASH-1 (for review see Huber (2006) and Rohrer (2011)). We show here that Islet-1 expression is absent in primary sympathetic ganglia of Phox2B deficient mice. This finding establishes Islet-1 as an additional member of the group of transcription factors acting downstream of Phox2B in the SA lineage. Islet-1 apparently does not influence the expression levels of Phox2A/B, Hand2, Mash-1 and Gata3 in sympathetic neurons and chromaffin cells.

However, according to our microarray data, we found three transcription factors among the gene products that were significantly down-regulated in adrenal glands of Islet-1^wnt1cre^ mouse embryos known to be associated with the development of SA cells, i.e. Gata2, AP2β, and Hand1. Out of these genes, Gata2 displayed the most pronounced alteration of its expression level (2.9 fold down) and we could confirm its down-regulation in sympathetic neurons and chromaffin cells of Islet-1^wnt1cre^ mouse embryos by in situ hybridization. In murine SA cells Gata2 is a direct or indirect downstream target of Gata3 with functions most likely identical to or at least overlapping with that of Gata3. In the chick, however, only Gata2 is present (Lim et al., 2000; Moriguchi et al., 2006; Tsarovina et al., 2004). Interestingly, Islet-1^wnt1cre^ mouse embryos and Gata3 knockout mice display some similarities with regard to the developmental deficits observed in sympathetic ganglia. This includes the timing and extent of sympathetic neuron death and the fact that the initial differentiation of SA cells is grossly normal, though, unlike in Islet-1^wnt1cre^ mice, the expression levels of TH were markedly reduced in sympathetic neurons and chromaffin cells of Gata3 mice (Moriguchi et al., 2006; Tsarovina et al., 2004). It should be noted that Gata transcription factors and Islet-1 have been shown to synergistically regulate some aspects of heart development (Dodou et al., 2004; Golzio et al., 2012). Together these observations point to the possibility that Islet-1 may co-regulate some functions of Gata3 during the development of SA cells.

### Functions of Islet-1 for the development of neuronal versus endocrine SA derivatives

Though sympathetic neurons and chromaffin cells share a common set of transcription factors during their early development, gene ablation studies have revealed distinct deficits in sympathetic neurons and chromaffin cells following the null mutation of various individual transcription factors (for review see Huber (2006) and Huber et al. (2009)). Therefore it was not surprising that the loss of Islet-1 affects neuronal and endocrine SA derivatives in a different way. Yet it should be noted that Islet-1 in general, like other LIM-HD proteins, appears to execute a very diverse array of functions depending on cellular context specific co-regulators (for review see Hobert and Westphal (2000)).

Sympathetic neurons, as we show here, undergo apoptosis in the absence of Islet-1 after having accomplished a grossly normal initial differentiation. How does Islet-1 deficiency affect sympathetic neurons survival? Islet-1 may simply interfere with the basic apoptotic machinery as suggested as a mode of action of Gata3 in maintaining the survival of mature sympathetic neurons (Tsarovina et al., 2010). Alternatively, cell death in Islet-1^wnt1cre^ mouse embryos may be the result of a lack of correct further differentiation of sympathetic neurons beyond the stage of a catecholaminergic neuroblast. The fact that the few sympathetic neurons that survive up to E16.5 in Islet-1^wnt1cre^ mouse embryos fail to correctly express the receptor tyrosine kinase TrkA and instead maintain the expression of c-Ret, a feature of early sympathetic neuroblasts, corroborates the latter notion. Nevertheless, we cannot exclude the possibility that the TrkA-negative and c-Ret-positive subpopulation of sympathetic neurons is specifically spared in Islet-1^wnt1cre^ mouse embryos. However, it should be noted that expression of TrkA in sympathetic ganglia is not initiated before E13.5 (Fagan et al., 1996), i.e. after the onset of substantial cell loss in sympathetic ganglia of Islet-1^wnt1cre^ mouse embryos.

The numerical deficits of chromaffin cells in Islet-1^wnt1cre^ mice were much less pronounced than that of sympathetic neurons at least up to E16.5. The most striking alteration that we found in the adrenal gland of Islet-1^wnt1cre^ mice was the virtual lack of PNMT expression. The distinct survival rates of sympathetic neurons and chromaffin cells in the absence of Islet-1 and the fact that in the adrenal gland primarily the expression of PNMT was affected may indicate that Islet-1 exerts fundamentally distinct functions in the two cell types. However, it is also conceivable that the molecular mechanisms underlying Islet-1 action is similar in sympathetic neurons and chromaffin cells, but leads to a distinct outcome depending on the cellular context. Different survival spans of intra-adrenal and extra-adrenal SA cells upon developmental impairment have been described before (for review see Huber (2006) and Huber et al. (2009)) and may be caused either by intrinsic differences between endocrine and neuronal SA cells or by a survival supporting environment provided by the adrenal gland.

The virtual lack of PNMT expression in the adrenal gland of Islet-1^wnt1cre^ mice could be due to an impairment of overall chromaffin cell differentiation to a point where PNMT expression can be enabled. However, based on their ultrastructure and gene expression profile chromaffin cells of Islet-1^wnt1cre^ mouse embryos appeared to have developed relatively normal in general, with some minor alterations. Furthermore, our microarray analysis did not reveal massive changes of the expression level of genes known to regulate PNMT expression, as e.g. the glucocorticoid receptor, Egr1 or AP-2 (for review see Wong (2003)). Thus it is also conceivable that Islet-1 may directly co-regulate PNMT expression or possibly interact with the glucocorticoid receptor, which is essentially required for PNMT expression in chromaffin cells (Finotto et al., 1999). It should be pointed out here, that physical interactions of Islet-1 with other nuclear steroid receptor have been described before (Gay et al., 2000).

In conclusion, our data show that Islet-1 is required for distinct aspects of the development of neuronal and endocrine SA lineage derivatives beyond the stage of a catecholaminergic neuroblast. In the future it will be intriguing to identify the mechanisms and co-regulators underlying Islet-1 function in cells of the SA lineage. Possible candidates include Gata transcription factors and the glucocorticoid receptor.

## Figures and Tables

**Table 1:** Gene expression profiling of adrenal glands of E14.5 control and Islet-1^wnt1cre^ mouse embryos by microarray analysis. Genes whose transcript expression levels were significantly changed (*p*<0.001) at least 2-fold and that are associated with chromaffin cell function or SA cell development were selected for display. The mean fold change (*n*=4 per group) is shown.

Gene symbol	Gene name	FC
*Hormone synthesis and secretion*		
PNMT	Phenylethanolamine-N-methyltransferase	−13.6
Rab3b	RAB3B, member RAS oncogene family	−2.3
Chga	Chromogranin A	−2.2
*Ion channels*		
Kcnmb2	Potassium large conductance calcium-activated channel, subfamily M, beta member 2	−2.0
*Transcription factors*		
Gata2	GATA binding protein 2	−2.9
Tcfap2b	Transcription factor AP-2 beta	−2.5
Hand1	Heart and neural crest derivatives expressed transcript 1	−2.0
*Receptors*		
Gfra3	Glial cell line derived neurotrophic factor family receptor alpha 3	−2.7

## Appendix A. Supplementary materials

Supplementary data associated with this article can be found in the online version at http://dx.doi.org/10.1016/j.ydbio.2013.04.027.
